# Socio-evolutionary role of bacterial membrane vesicles in cooperation and competition within microbial communities

**DOI:** 10.1093/ismejo/wrag165

**Published:** 2026-06-25

**Authors:** Zongping Li, Qiwen Yang

**Affiliations:** Department of Laboratory Medicine, Peking Union Medical College Hospital, Chinese Academy of Medical Sciences and Peking Union Medical College, Beijing 100730, China; Graduate School, Peking Union Medical College, Chinese Academy of Medical Sciences, Beijing 100730, China; Department of Laboratory Medicine, Peking Union Medical College Hospital, Chinese Academy of Medical Sciences and Peking Union Medical College, Beijing 100730, China; Key Laboratory of Pathogen Infection Prevention and Control (Peking Union Medical College), Ministry of Education, Beijing 100730, China

**Keywords:** bacterial membrane vesicles, sociomicrobiology, social evolution, cooperation, competition, public goods, bacterial community, microbial interaction

## Abstract

Microbial communities are shaped by social interactions that influence survival, resource access, and evolutionary trajectories. Most studies of microbial social evolution have focused on freely diffusible molecules such as siderophores and quorum-sensing signals, whereas the physical form in which extracellular products are delivered has received less attention. Bacterial membrane vesicles package enzymes, lipids, nucleic acids, and small metabolites into membrane particles. Membrane packaging slows cargo dispersal, protects labile cargo, and can restrict uptake through receptor-dependent binding. Here, we review vesicle-mediated interactions from the perspective of social evolution, with a primary focus on outer membrane vesicles released by gram-negative bacteria and drawing on gram-positive examples where informative. We first examine how the route of vesicle biogenesis influences cargo composition and cost to the producing cell, and consider how these differences may influence ecological function. We then discuss how limited dispersal and recipient bias may reduce benefit leakage and thereby help stabilize cooperation. Finally, we consider the conditions under which vesicles support antagonistic outcomes, including toxin delivery, lytic activity, and resource piracy. We argue that whether vesicles favour cooperation, privatization, or competition is not a fixed property but depends on the interplay among biogenesis route, the surface receptor repertoire of recipient cells, local relatedness, and environmental conditions including resource availability and niche overlap. We further highlight that bulk vesicle preparations confound analysis of functionally distinct vesicle subpopulations, underscoring the need to resolve vesicle type, biogenesis route, and recipient identity in spatially structured communities.

## Introduction

Microorganisms typically live in dense, multispecies communities in which neighbouring cells profoundly influence one another’s survival, fitness, and evolutionary trajectories [1, 2]. Research in sociomicrobiology has shown that bacteria display diverse social behaviours, including cooperation, competition, cheating, and kin discrimination [3]. Many of these behaviours are mediated by extracellular products that function as public goods, that is, resources that are costly to produce but can benefit surrounding cells [1, 4, 5]. This raises a central problem in microbial social evolution: how can public goods persist when they are vulnerable to exploitation by non-producing cheaters? Hamilton’s rule (*rb > c*) [6–8] provides one way to analyse this problem by asking whether the benefit *(b)* returned to genetically related recipients is large enough to offset the cost *(c)* paid by the producer. Most experimental and theoretical studies have examined freely diffusible molecules, such as siderophores and quorum-sensing signals [9, 10], but the physical form of the released product can also influence who receives the benefit and how far that benefit spreads.

Bacterial membrane vesicles (BMVs) differ from freely diffusible molecules because they package cargo into lipid particles [11]. As particulate structures with limited diffusion and surface receptor specificity, they can concentrate benefits among nearby related cells, reducing exploitation by non-producing neighbours, thereby helping to maintain cooperative interactions [12, 13]. Yet the same delivery system can also mediate competition by transporting lytic enzymes, toxins, and nutrient acquisition systems that damage rivals or capture limiting resources [14, 15]. These contrasting social outcomes may be influenced in part by the route of vesicle biogenesis. Two routes are particularly relevant for social evolution: membrane blebbing in living cells and endolysin-triggered cell lysis. Vesicles produced by each route differ in cargo composition, cost to the producing cell, and probable ecological function [16, 17].

This review asks how vesicle features may influence the social outcome of vesicle release. We focus on three linked questions. First, how do biogenesis routes influence cargo composition and producer cost? Second, how do limited vesicle movement, biofilm retention, and receptor-dependent uptake restrict access to vesicle-associated benefits? Third, under which conditions do vesicles act as shared protective particles, restricted-access resources, or competitive agents that damage rivals or capture limiting nutrients? We focus primarily on outer membrane vesicles (OMVs) from gram-negative bacteria and include gram-positive examples when they clarify common principles. By organizing BMV functions around producer cost, cargo composition, recipient identity, and spatial population structure, we aim to provide a framework for understanding how vesicle-mediated interactions can support shared protection, restricted access to resources, or competition in different microbial settings. We also highlight vesicle heterogeneity as an important unresolved issue because bulk vesicle preparations may combine particles that differ in size, cargo, biogenesis route, and biological activity.

## Biogenesis routes influence the ecological properties of BMVs

### Biogenesis of bacterial membrane vesicles

The formation of BMVs is a complex process driven by multiple factors. Current studies support two basic routes, membrane blebbing from living cells and endolysin-triggered cell lysis [17, 18].

#### Membrane blebbing

Membrane blebbing is the classical route of BMVs and occurs in living cells without causing cell death. In gram-negative bacteria, it occurs when local coupling between the outer membrane and the peptidoglycan layer is weakened, allowing the outer membrane to protrude outward and pinch off as OMVs [16]. Several molecular mechanisms that promote blebbing have been identified.

**Figure 1:**
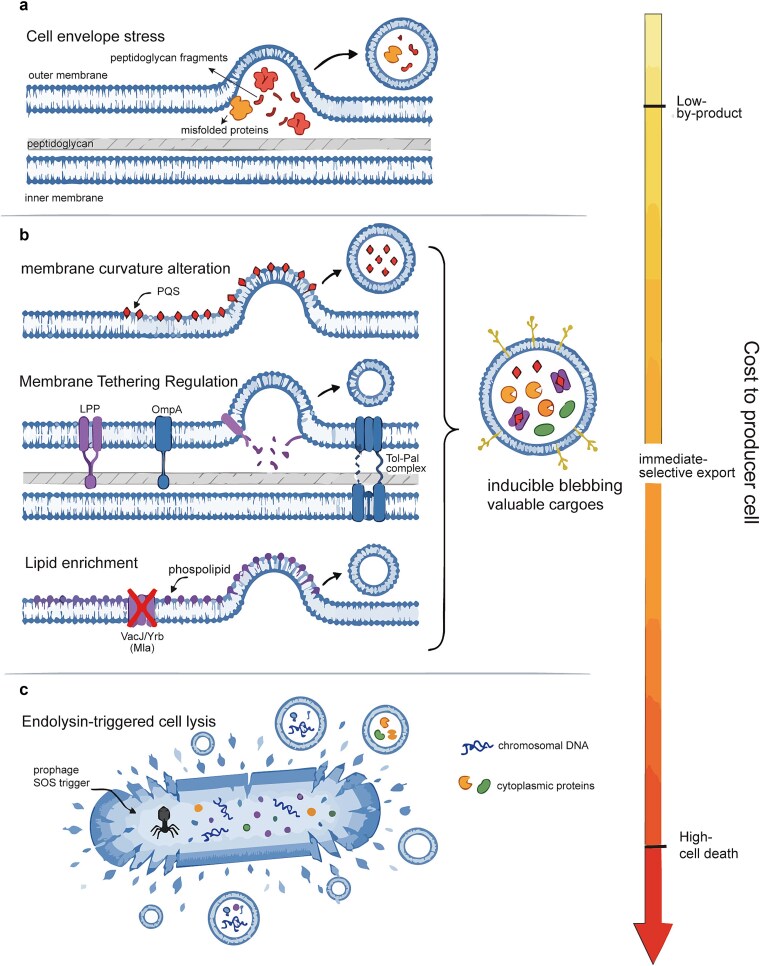
Mechanisms of BMV biogenesis, molecular composition, and associated producer costs. (a) Cell envelope stress results from periplasmic accumulation of misfolded proteins or peptidoglycan fragments, disturbing envelope homeostasis and promoting outward bulging of the outer membrane. (b) Three major mechanisms of inducible blebbing in living cells are illustrated: membrane curvature alteration, which involves the insertion of small hydrophobic molecules (e.g. PQS) into the outer leaflet to induce curvature; membrane tethering regulation, which involves the weakening of linkages (e.g. Lpp, OmpA, Tol-Pal) between the outer membrane and peptidoglycan layer; and lipid enrichment, which involves disrupted phospholipid asymmetry (e.g. loss of VacJ/Yrb) leading to phospholipid accumulation in the outer leaflet. (c) Endolysin-triggered cell lysis involves prophage-induced degradation of the peptidoglycan layer, cell rupture under turgor pressure, and reassembly of membrane fragments into BMVs that entrap cytoplasmic contents.

Envelope stress is one important biogenesis mechanism. Accumulation of misfolded proteins, peptidoglycan fragments, or abnormal lipopolysaccharide (LPS) in the periplasm disturbs envelope homeostasis and promotes local outward bulging [19] (Fig. 1a). Reduced membrane tethering can also drive blebbing. The outer membrane is normally linked to peptidoglycan by structures such as Braun lipoprotein (Lpp), outer membrane protein A (OmpA), and the Tol-Pal complex [20]. When these connections are weakened, vesiculation increases [21, 22]. In addition, small hydrophobic molecules can remodel membrane curvature. The best-known example is *Pseudomonas* quinolone signal (PQS), which inserts into the outer leaflet and stimulates OMV formation [23]. Disruption of phospholipid homeostasis provides another mechanism. The VacJ/Yrb transport system normally maintains outer membrane lipid asymmetry by removing phospholipids from the outer leaflet. When this system is mutated or repressed by ferric uptake regulator (Fur) under iron limitation, phospholipids accumulate in the outer leaflet and promote vesicle release [24] (Fig. 1b). Together, these findings indicate that blebbing is not a single fixed mechanism, but a flexible outcome of envelope remodeling.

#### Endolysin-triggered cell lysis

Recent studies have identified a distinct route of BMV formation in which bacteriophage-derived endolysins degrade peptidoglycan and trigger cell death [17, 25].

In gram-negative bacteria, DNA damage can activate the SOS response and induce prophage-encoded holin-endolysin systems. Holin forms pores in the cytoplasmic membrane, allowing endolysin to enter the periplasm and degrade the peptidoglycan layer [17, 26]. The cell then ruptures under turgor pressure, and the shattered membrane fragments curl and reassemble into explosive outer membrane vesicles (EOMVs) and outer-inner membrane vesicles (OIMVs) [17, 27]. This process is termed ‘explosive cell lysis’ (ECL) (Fig. 1c). Unlike blebbing-derived OMVs, these vesicles can trap cytoplasmic proteins and chromosomal DNA during reassembly [17].

In gram-positive bacteria, the core trigger is similar, but the structural outcome differs. In *Bacillus subtilis* [25], endolysin encoded by the defective prophage PBSX, a *B. subtilis* phage-like element, opens holes in the peptidoglycan, through which the cytoplasmic membrane protrudes and forms vesicles. Because the thick cell wall is not completely destroyed, the cell retains its overall cellular morphology but loses membrane integrity and dies, generating ghost cells and intracellular vesicles. This process has been described as ‘bubbling cell death’.

Multiple environmental cues can promote vesicle production through different biogenesis routes. Elevated temperature, NaCl-induced hyperosmotic stress, polymyxin, and non-lethal H₂O₂ exposure have been associated with increased OMV release or altered OMV cargo, likely through envelope perturbation and membrane remodeling [28–31]. In contrast, DNA-damaging stresses such as ultraviolet radiation and ciprofloxacin can activate SOS- and prophage/endolysin-associated pathways, promoting lysis-derived vesicle formation [17, 32]. Pyocyanin-mediated H₂O₂ generation may further link oxidative stress to lysis-associated outcomes, including cell lysis, extracellular DNA (eDNA) release, and increased BMV formation [33].

### Why the route of biogenesis matters for social evolution

Blebbing and endolysin-triggered lysis generate vesicles that differ in cargo composition, producer cost, and may also differ in ecological role.

#### Biogenesis route influences cargo composition

The route of biogenesis strongly influences vesicle cargo. Blebbing-derived OMVs usually originate from the outer membrane and periplasm and therefore are enriched in outer membrane lipids, lipopolysaccharide, and periplasmic components [16, 18, 34]. Their cargo is not always random. In *Bacteroides thetaiotaomicron* (*B. thetaiotaomicron*), specific export signals direct enzymes into OMVs, and OMV protein composition changes with the available polysaccharide substrate [35]. In enterotoxigenic *Escherichia coli* (*E. coli*), oxidized proteins are preferentially packaged into OMVs during oxidative stress [31]. In *Pseudomonas aeruginosa* (*P. aeruginosa*), PQS not only induces OMV formation but is also loaded into vesicles for signal delivery [36]. Beyond PQS, vesicle-associated delivery has also been reported for hydrophobic small molecules such as CAI-1 in *Vibrio harveyi* [37] and violacein in *Chromobacterium violaceum* [38], the latter of which promotes OMV formation for its own export. This selective loading capacity enables blebbing-derived OMVs to support distinct functions, including signal dissemination, antibiotic decoy activity, phage sequestration, and predatory interactions [39–41]. However, these functions are not exclusive to blebbing-derived vesicles. Lysis-derived vesicles that contain similar cargoes can also perform these roles, and sometimes more efficiently as shown by enhanced quorum-sensing signal release through endolysin-dependent vesicle formation in *Paracoccus denitrificans* [42]. Furthermore, lysis-derived vesicles are formed from fragmented membrane sheets during cell rupture and can entrap cytoplasmic proteins, DNA, and inner membrane components [17, 18]. Their cargo profile also links them closely to horizontal gene transfer, biofilm matrix construction, and the release of multiple extracellular factors [17, 43].

#### Biogenesis route influences producer cost

From the perspective of social evolution, the cost paid by the vesicle-producing cell, the *c* term in Hamilton’s rule (*rb > c*), may be the variable most strongly affected by the route of biogenesis.

When BMV release primarily functions to relieve envelope stress, vesiculation acts as a way to remove harmful materials [44]. In this case, any benefit to neighbouring cells is closer to a by-product benefit than to a dedicated cooperation [45]. In contrast, inducible cargo-enriched vesiculation can export molecules that are still useful to the producing cell, such as β-lactamases during β-lactam exposure [46, 47] or glycoside hydrolases during polysaccharide utilization [35]. This process is therefore not simply waste removal, but also involves the loss of functional enzymes that could support producer survival, defence, or nutrient acquisition. The cost is highest in lysis-derived vesiculation, where the producer may lose membrane integrity or die altogether. Cell lysis additionally co-releases eDNA, phages, and pyocins alongside vesicles, which contribute to biofilm matrix formation, horizontal gene transfer, and interference competition. Such a trait is difficult to explain through direct benefit alone and fits more naturally within a kin selection framework, in which high relatedness and spatial confinement help return benefits to close relatives [3].

## Properties that make BMVs ecologically distinct from freely diffusible molecules

**Figure 2:**
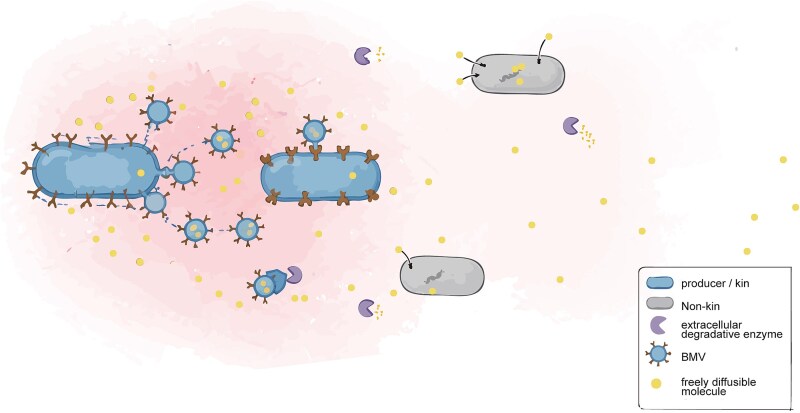
Dispersal of BMVs compared with freely diffusible molecules. A producer cell releases both BMVs and freely diffusible molecules. BMVs travel over limited distances and remain associated with the producing colony, where receptor-mediated recognition restricts productive uptake largely to clonal neighbours (kin). Freely diffusible molecules disperse broadly into the surrounding environment, where they can reach non-kin cells or be degraded by extracellular enzymes before reaching a recipient.

Compared with freely diffusible molecules, BMVs are particulate structures whose movement in the extracellular environment is constrained by their size, surface-associated ligands, and interactions with substrata. Real-time tracking experiments demonstrated that BMVs travel substantially slower than soluble compounds and frequently move by surface-associated sliding rather than free diffusion [48] (Fig. 2). In biofilms, this constraint is amplified, because BMVs can be retained within the extracellular polymeric matrix [49]. Membrane encapsulation can additionally shield labile cargo such as enzymes and signalling molecules from extracellular degradation, increasing the probability that delivered material remains functional at the site of uptake.

BMV uptake is not uniform across recipient cells; this selectivity may reflect surface molecule recognition and physicochemical compatibility between vesicles and recipients. In *Buttiauxella agrestis*, OMVs associate predominantly with conspecific cells through outer membrane protein recognition and lower interaction energy [12], which means even an OMV that physically reaches a non-clonemate may not be productively taken up.

The limited dispersal and recipient selectivity of BMVs are not absolute. Antibiotic treatment that reduces the density of cell-surface appendages increases OMV velocity and total displacement [48]. Similarly, phylogenetically distant competitors can intercept OMVs by deploying their own surface receptors that recognize broadly conserved vesicle ligands such as LPS [15]. The effective dispersal distance and functional range of OMVs are therefore subject to modulation by both environmental conditions and the receptor repertoires of co-resident cells.

## Vesicle-mediated cooperation: shared defence and its evolutionary stabilization

BMVs are increasingly recognized as social traits whose benefits can extend to neighbouring cells, raising the question of when vesicle release qualifies as cooperation in the evolutionary sense.

### Modes of vesicle-mediated shared protection

#### Enzymatic shields

**Figure 3:**
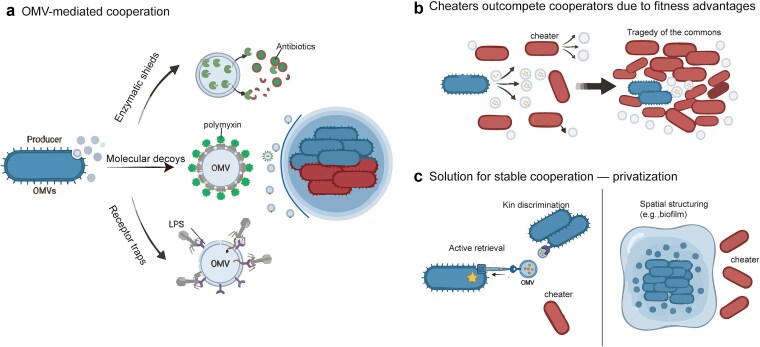
BMV-mediated cooperation and stabilization mechanisms. (a) Vesicle-mediated cooperation operates through three primary modes: enzymatic shields, where vesicles carry enzymes (e.g. β-lactamases) to hydrolyze extracellular antibiotics; molecular decoys, which adsorb membrane-active agents to protect the producer and neighbours; receptor traps, which sequester bacteriophages through parental surface receptors to provide community-level antiphage defences. (b) Cheaters, defined as cells producing fewer or non-functional BMVs, exploit the shared protection provided by cooperators without paying the corresponding production cost; the resulting fitness advantage may eventually collapse the cooperative system, leading to the tragedy of the commons. (c) Solutions for stable cooperation through privatization: active retrieval and kin discrimination involve producer-encoded surface receptors that selectively recapture BMVs and their cargo, returning benefits to clonemates. Spatial structuring (e.g. biofilm) involves clonal microcolonies that create physical diffusion barriers, confining BMVs within high-relatedness neighbourhoods and excluding unrelated cheaters.

One of the most direct cooperative functions of BMVs is the delivery of antibiotic-degrading enzymes into the shared extracellular space (Fig. 3a). Resistant bacteria can package enzymes such as β-lactamases into BMVs, allowing vesicles to hydrolyze antibiotics outside the cell and thereby protect both producers and nearby susceptible neighbours. This effect has been reported in multiple systems, including NDM-5-carrying OMVs from *E. coli* [47] and TEM β-lactamase-containing OMVs from *Neisseria gonorrhoeae* [46], and similar protection has also been reported in polymicrobial and interspecies settings [50, 51]. Such enzymatic shields are socially significant because they create a local zone of antibiotic detoxification that benefits neighbouring cells, rather than confining protection to the producing cell alone. Incomplete detoxification can create sub-inhibitory antibiotic gradients, which select for stable outer membrane porin D (OprD) mutations in susceptible *P. aeruginosa*, highlighting the long-term evolutionary impact of this cooperative behaviour [52].

#### Molecular decoys

Under attack by membrane-active agents such as polymyxins, bacteria often increase BMV production [28, 40], and these vesicles can function as decoys by adsorbing antimicrobial agents onto membrane surfaces. In *E. coli*, OMVs protect neighbouring cells against polymyxin but not against antibiotics with unrelated intracellular targets via this decoy mechanism [53], which relies on direct membrane adsorption rather than enzymatic degradation. Further investigations have revealed that the molecular basis of OMV binding efficacy depends primarily on their lipid composition. Increased OMV production can expand the total lipid A surface available for polymyxin binding [28], whereas lipid A modification may weaken colistin adsorption [54]. Socially, these vesicles matter because they absorb membrane-active agents into particulate structures that can protect nearby cells without requiring direct sacrifice of viable cells.

#### Receptor traps

Bearing identical surface receptors (e.g. LPS and outer membrane proteins) to the vesicle-producing cells, BMVs competitively adsorb free phage particles, neutralizing them before they can infect viable bacteria [40, 41, 55]. This receptor-trap mechanism has been reported in *E. coli, Vibrio cholerae*, and *P. aeruginosa*, with efficacy depending on receptor abundance such as LPS O1 antigen, OmpU, or phage-specific LPS structures [40, 41, 56, 57]. This OMV-mediated mechanism thus constitutes a form of community-level antiphage defences [40], representing a low-cost strategy for individual cells (sacrificing vesicles rather than undergoing lysis). It has also been reported that lysis-derived OMVs exhibit stronger phage-neutralizing activity than blebbing-derived OMVs [58]. However, whether this phenomenon extends specifically to ECL-derived vesicles remains to be established through direct comparative evidence. This raises the possibility that the most defensive vesicles may also be those whose production is most costly to the producing cell, a trade-off we revisit under “Spatial structuring and limited dispersal”.

### From by-product benefit to altruism: when shared protection requires evolutionary explanation

Vesicle-mediated defence can protect more than the vesicle-producing cell. However, from a social evolution perspective, any trait in which some individuals pay a cost to produce a benefit shared with neighbours is vulnerable to cheating [59]: bacteria that contribute fewer or non-protective BMVs avoid the cost but still gain from collective protection, thereby gaining a growth advantage. Such free riding can generate persistent evolutionary conflict. As the proportion of low-contributing individuals increases, the relative fitness of cooperators declines, which may eventually cause the cooperative system to collapse, a situation referred to as ‘the tragedy of the commons’ [60] (Fig. 3b). This leads to a central question: what makes vesicle-mediated cooperation evolutionarily stable in the face of potential cheater invasion?

Before discussing solutions to the problem of cooperation, it is useful to distinguish whether a cooperative behaviour is mutually beneficial or altruistic. This distinction is important because the two situations demand a different set of explanations. In principle, explanations for the evolutionary stability of cooperation fall into two categories [61]. First, the cooperative behaviour can provide a direct fitness benefit to the producing cell that outweighs the cost of vesicle production. In this case, cooperation is mutually beneficial and requires no special explanation. Second, and more challenging to account for evolutionarily, are cooperative behaviours that reduce the direct fitness of the actor. In this case, cooperation is altruistic and can only be maintained if the benefits are directed toward individuals that share the genes underlying vesicle production; this is kin selection, which provides an indirect fitness benefit [1].

Applying the distinction between direct and indirect fitness requires first asking whether vesicle release provides a direct benefit to the producer or benefits neighbours at the producer’s expense. When vesiculation primarily functions to relieve envelope stress by removing misfolded proteins or aberrant material, the producing cell gains a direct fitness benefit irrespective of the effect on neighbours. Strictly speaking, under these conditions, vesicle release would not be classed as cooperation because the benefit to neighbouring cells is simply the by-product of a stress-relief behaviour. A behaviour should only be classed as cooperative if it is maintained, at least partly, because of its beneficial effect on the recipient [1, 61]. However, cooperation can evolve in response to such by-product benefits. If stress-released vesicles reciprocally protect neighbouring cells, selection can favour increased vesicle production beyond the level needed for stress relief alone, because a cell that better protects its neighbours also receives greater protection in return. Whether vesicle-mediated shared protection in any system has reached this stage remains an open empirical question.

The evolutionary problem becomes more acute when vesicle production is inducibly upregulated and coupled to the selective loading of protective cargo. Many such vesicle-mediated traits are whole-group traits that can provide both a direct and indirect fitness benefit [7, 61]. Vesicle-derived enzymatic shields, for example, protect both the producing cell and nearby relatives, so whether such a trait is mutually beneficial or altruistic depends on the relative magnitude of direct and indirect returns, which will vary with population density, spatial structure, and the scale over which vesicle cargo disperses. By contrast, endolysin-triggered cell lysis is an other-only trait [7, 61]: the lysed cell gains no direct benefit [17], and maintenance of this behaviour therefore depends on high local relatedness and spatial confinement that channel benefits to clonemates [62]. It is primarily the altruistic cooperation that demands explanation, and in the following section, we discuss the mechanisms through which it can be stabilized.

### Stabilization of altruistic vesicle-mediated cooperation

A general principle from social evolution theory is that cooperation is more stable when benefits remain close to the producer and its relatives [63, 64]. The properties of BMVs, particularly limited dispersal and receptor-mediated binding, are therefore directly relevant to the stability of vesicle-mediated cooperation. In the following subsections, we examine two mechanisms that build on these properties: spatial structuring and active retrieval with kin-biased access.

#### Spatial structuring and limited dispersal

Clonal spatial confinement within structured environments such as biofilms is the most widespread mechanism for stabilizing vesicle-mediated cooperation. When biofilms are composed of clonal microcolonies, the local neighbourhood of a producing cell is dominated by close kin (high effective *r*) [65]. In this setting, the particulate nature of BMVs limits dispersal relative to freely diffusible goods. This spatial limitation confines the benefits within kin groups, drastically reducing leakage to unrelated potential cheaters in distant microcolonies [13].

Spatial structure is even more critical for endolysin-triggered cell lysis, the most extreme form of vesicle release. Cell lysis has been most clearly documented in biofilms and surface-attached communities in *P. aeruginosa*, whereas spontaneous lysis appears much rarer in planktonic cultures [17, 26]. This distribution is socially important because biofilms should retain vesicles and eDNA near related neighbours rather than allowing them to disperse broadly. In addition, ECL occurs in only a small subpopulation of cells [17]. The OMVs generated during lysis subsequently shield surviving relatives against a broad range of envelope-targeting threats, including phages, membrane-active antibiotics, and eukaryotic antimicrobial factors [28, 40, 58]. Because the cost of lysis is borne by a minority, whereas protection is distributed across a kin-dominated neighbourhood, biofilm-scale spatial confinement maximizes the indirect fitness returns of this extreme form of vesicle release.

#### Active retrieval and kin discrimination

Restricted access to vesicle-associated benefits provides another mechanism for stabilizing vesicle-mediated cooperation. In this case, BMVs no longer behave as fully open public goods, but instead as restricted-access or club-like goods. A well-studied example comes from *P. aeruginosa*, where the type VI secretion system (T6SS) effector TseF associates with PQS-containing OMVs and promotes their retrieval through specific surface receptors [15, 66]. This coupling helps return iron-associated vesicle cargo to the producer lineage rather than allowing indiscriminate diffusion to unrelated competitors. A related principle is seen in *B. thetaiotaomicron*, where the OMV-surface lipoprotein XusB binds enterobactin and enables its utilization by *B. thetaiotaomicron* and other *Bacteroidetes* encoding XusB homologs [67]. Similar kin-restriction is observed in gram-positive bacteria, such as *Dietzia* sp. DQ12-45-1b, where iron-laden membrane vesicles promote growth within the order *Corynebacteriales* but are inaccessible to distantly related species like *E. coli* or *Pseudomonas* spp. [68].

Spatial structuring and active retrieval provide complementary routes to stabilizing OMV-mediated cooperation in structured communities (Fig. 3c). Yet under different ecological conditions, OMVs can also act as competitive agents, as we show next.

## Competitive OMVs: weapons of interference and exploitation

Competition occurs when a phenotype in one strain causes a fitness decrease in another strain competing for the same resources [69]. In microbial communities, this usually requires overlap in resource use, which distinguishes competition from predation and parasitism. Whereas predators or parasites exploit another organism as a resource, competing microbes depend on the same external resources, such as nutrients (limiting sources of carbon, nitrogen, phosphorus, as well as trace elements such as iron) and space (favourable surfaces and microenvironments, including mucosal surfaces, plant roots, soil aggregates, and positions within biofilms).

**Figure 4:**
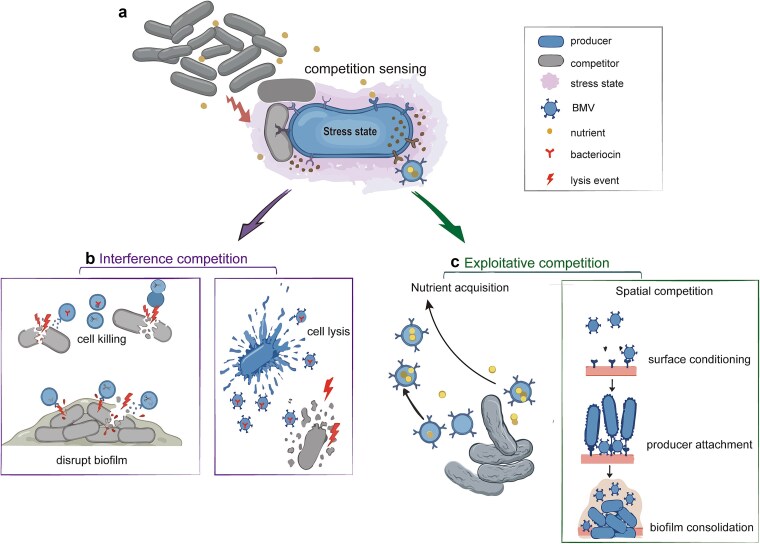
BMV-mediated competitive strategies driven by competition sensing. (a) Competition sensing: bacteria interpret environmental stressors, such as nutrient limitation, membrane damage, or the presence of rivals, as signals of ecological competition. These cues trigger a stress state that upregulates antagonistic traits mediated by BMVs. (b) Interference competition: vesicle-delivered peptidoglycan hydrolases and bacteriocins fuse with the envelopes of competitors, mediating cell killing and disrupting established biofilms. Cell lysis amplifies this effect by releasing vesicle-associated lytic factors that damage surrounding rivals. (c) Exploitative competition: exploitative competition involves two strategies. Nutrient acquisition: BMVs enriched with specialized receptors capture limiting nutrients (e.g. iron) from the environment, depriving competitors. Spatial competition: BMVs participate in surface conditioning, producer attachment, and biofilm consolidation, allowing the producer lineage to monopolize favourable niches.

Bacteria adjust the composition and production of BMVs in response to ecological cues, transforming these vesicles from cooperative vehicles into competitive weapons to maximize fitness. This aligns with the ‘competition sensing’ hypothesis [3, 70], which posits that bacteria interpret nutrient limitation or cellular damage as signals of ecological competition and upregulate traits that improve resource capture or suppress rivals [71]. Indeed, contemporary research confirms that BMVs are a crucial component of these plastic competitive responses [72]. For instance, membrane damage by polymyxin increases OMV biogenesis as a defence mechanism [28], and *P. aeruginosa* elevates OMV release under diverse stresses including oxidative stress [29] (Fig. 4a).

Under conditions of strong niche overlap [70], BMV function pivots toward aggression and nutrient acquisition primarily through two strategies: (i) interference competition (Fig. 4b), in which an individual reduces the fitness of its competitors through direct damage, displacement, or suppression, and (ii) exploitative competition (Fig. 4c), in which an individual reduces the resources available to its competitors by acquiring those resources more rapidly or more efficiently, without direct contact with the competitor.

### Interference competition

One well-characterized form of vesicle-mediated interference is the delivery of envelope-damaging enzymes. *P. aeruginosa* OMVs carry peptidoglycan hydrolases that degrade the cell walls of both gram-negative and gram-positive bacteria through membrane fusion or peptidoglycan adhesion [14, 73]. Under aminoglycoside exposure, antibiotics may be co-packaged with these hydrolases, enhancing potency [14]. Similar OMV-mediated bacteriolytic activity has been reported in at least eight gram-negative genera [74], indicating that localized enzymatic attack is widespread.

Bacteriocins, narrow-spectrum protein toxins that kill close relatives of the producer, which is itself protected by linked immunity genes [69, 75, 76], can also be vesicle-delivered [77]. In *Lactobacillus acidophilus* [77], induction of Lactacin B enriches secreted membrane vesicles with putative bacteriocins encoded by the *lab* operon. These vesicles inhibit the growth of *Lactobacillus delbrueckii* and damage its membrane integrity, providing direct evidence of vesicle-borne bacteriocin delivery in gram-positive bacteria. Vesicles may also be essential for the activity of some hydrophobic bacteriocins. For example, micrococcin P1 has low water solubility, and its antibacterial activity relies on vesicle binding to reach and fuse with target cytoplasmic membranes [78].

BMVs can also mediate interference competition by disrupting established biofilms. For instance*, Burkholderia thailandensis* OMVs reduce viable counts, biomass, thickness, and structural integrity of *Streptococcus mutans* (*S. mutans*) biofilms [79]. In *P. aeruginosa*, OMV-associated leucine aminopeptidase detaches non-self *P. aeruginosa* and *Klebsiella pneumoniae* biofilms [80], and *Lactobacillus gasseri* vesicles disrupt *Gardnerella vaginalis* and *Staphylococcus aureus* biofilms [81].

ECL likewise contributes to interference. In *P. aeruginosa*, the same lytic pathway releases R- and F-type pyocins, phage-tail-like bacteriocins that travel tens of micrometres and kill nonclonal conspecifics [82, 83], whereas producing cells and clonemates are shielded by their shared O-antigen serotype [82, 84]. ECL-derived OMVs additionally carry endolysins and peptidoglycan hydrolases [85], which may further enhance local lytic activity. However, current studies have rarely distinguished vesicles by biogenesis route, and whether cell lysis specifically increases the competitive efficacy of vesicles remains to be tested directly.

### Exploitative competition

Exploitative competition reduces competitor growth by depleting shared resources or pre-empting spaces, rather than inflicting damage.

#### Nutrient acquisition

Iron acquisition provides the clearest example of vesicle-mediated exploitative competition. In *Acinetobacter baumannii* (*A. baumannii*), OMVs enriched in TonB-dependent receptors bind heterologous siderophore complexes, including enterobactin produced by *E. coli*. These vesicles capture iron-loaded enterobactin in the extracellular environment and return the bound iron to the producer cell through membrane fusion, a process described as ‘xenosiderophore piracy’ [86]. A related strategy has been reported in *Cupriavidus necator* (*C. necator*) [15], where the T6SS effector TeoL binds LPS on extracellular OMVs, and the TeoL-OMV complex is tethered to producer cells through the receptors CubA and CstR for internalization. Because LPS is ubiquitous on gram-negative OMVs, this allows *C. necator* to recruit vesicles from phylogenetically distant neighbours, conferring advantages in iron acquisition, interbacterial competition, and horizontal gene transfer [15]. Heme acquisition by OMVs follows a similar receptor-mediated strategy. In *Porphyromonas gingivalis*, the hemophore-like HmuY sequesters heme and delivers it to the TonB-dependent receptor HmuR [87, 88]; its superior efficiency relative to homologous systems in cohabiting oral bacteria may confer a competitive advantage under heme limitation [89].

Receptor-mediated nutrient acquisition illustrates a common principle: when a nutrient is released as a soluble factor, access is determined by diffusion and uptake capacity, and any nearby cell can benefit. When the same acquisition function is carried out by a surface receptor on a vesicle, access is restricted to cells bearing compatible uptake machinery. This shift converts a broadly available resource into one selectively accessible to genetically related or physiologically compatible recipients.

#### Spatial competition

Space is another limiting resource in microbial communities. In biofilms, surface position determines nutrient access and exposure to competitors. BMVs can influence this process by conditioning surfaces, promoting producer attachment, or suppressing competitor biofilm formation. In *P. aeruginosa*, OMVs induced by mucosal components such as lysozyme prime epithelial surfaces for bacterial adhesion, increasing attachment efficiency approximately fourfold [90], suggesting that OMV release ahead of an advancing population may prime favourable niches for producer colonization. In addition, OMVs can further consolidate the producer’s position. In *Aeromonas veronii*, OMVs enhance biofilm formation dose-dependently [91]. Similarly, in *Helicobacter pylori* TK1402, OMVs are embedded in the biofilm matrix, associate with strong biofilm formation, and stimulate biofilm development when added exogenously [92]. More direct evidence for vesicle-mediated spatial competition comes from oral biofilms [93]. *Streptococcus gordonii* and *Streptococcus sanguinis* are the primary initial colonizers of tooth surfaces and compete with *S. mutans* for the same spatial niche. *S. mutans* membrane vesicles, which are enriched in glucosyltransferases (Gtfs), selectively suppress biofilm formation by both competitors without affecting their planktonic growth, indicating that the competitive effect targets surface colonization specifically.

## Conclusions and perspectives

Vesicle-mediated interactions can lead to protection, nutrient acquisition, signalling, or antagonism, depending on the cells that produce and receive the vesicles and on the conditions under which vesicles are released. Receptor-dependent uptake can bias access to vesicle-associated resources towards cells that carry the appropriate uptake machinery [66, 67]. By contrast, interactions with broadly distributed surface molecules, such as LPS, can allow *C. necator* to recruit OMVs from phylogenetically distant donors [15]. Similarly, vesicles carrying β-lactamases can protect nearby susceptible cells [46, 50, 51], whereas vesicles with bacteriolytic activities can damage non-self bacteria [14, 73, 77, 78]. The OMVs isolated from different developmental stages of biofilms have opposite effects on newly formed biofilms [94]. Cell lysis releases vesicles that may shield surviving relatives from phages and host antimicrobials and releases R- and F-type pyocins that kill nonclonal conspecifics [40, 95]. Thus, the outcome of vesicle exchange is not determined by cargo alone. It also depends on surface receptor repertoire of recipient cells, local relatedness, the spatial structure of the population, and prevailing environmental conditions including resource availability and niche overlap.

A remaining question is whether BMVs released by bacteria are functionally homogeneous. Increasing evidence suggests that this is not the case. *Pseudomonas putida* produces small vesicles of ~100 nm that carry catabolic enzymes, whereas larger vesicles appear mainly during late stationary phase and are enriched in outer-membrane proteins [96]. In *Aggregatibacter actinomycetemcomitans* JP2, the leukotoxin LtxA is associated with a larger OMV subpopulation, whereas the smaller and more abundant OMVs lack this toxin [97]. These observations suggest that growth phase and nutrient availability can alter the relative abundance of distinct vesicle types, which has important consequences for the interpretation of vesicle-mediated social effects. Most functional assays are performed with bulk vesicles, which combine vesicles that may differ in size, cargo, and biological activity. Such assays therefore report the net effect of a mixed population. Future studies should separate vesicles by size, density, or affinity and combine this fractionation with cargo proteomics and functional assays. Single-vesicle methods, including nano-flow cytometry and high-resolution imaging, will be needed to revisit conclusions that were originally drawn from bulk measurements.

The relationship between the biogenesis route and vesicle function also remains unresolved. A single species can release vesicles by membrane blebbing in living cells and by endolysin-triggered cell lysis. These two routes generate vesicles that differ most clearly in structure and composition. Blebbing-derived OMVs are mainly associated with outer-membrane and periplasmic components. Lysis-derived vesicles additionally contain cytoplasmic proteins, chromosomal DNA, endolysins, and phage-tail-like bacteriocins. It would be particularly valuable for future work to quantify the abundance of each vesicle type in natural environments and to elucidate which functions are preferentially associated with a particular biogenesis route.

Whether the social dynamics of BMV release and uptake can be resolved in natural communities is another open question. Most evidence for vesicle-mediated cooperation or competition comes from planktonic cultures and purified vesicle preparations. These systems are experimentally tractable, but they do not fully capture the spatial structure that determines which cells encounter vesicles in natural communities. Recent advances in spatially resolved single-cell transcriptomics, including parallel sequential fluorescence *in situ* hybridization (par-seqFISH) [98], and live fluorescent tracking of individual vesicles [48] now provide a way to connect vesicle production with local cell identity and vesicle movement. These approaches could be used to map where vesicle-producing cells occur, how far vesicles disperse, and which neighbouring cells they contact in structured communities or host-associated environments. Together, resolving vesicle heterogeneity, biogenesis route, and recipient identity will be essential for understanding how BMVs shape cooperation and conflict in natural microbial communities.

## Data Availability

Data sharing is not applicable to this article as no datasets were generated or analysed during the current study.
